# Value Alignment and Public Perceived Legitimacy of the European Union and the Court of Justice

**DOI:** 10.3389/fpsyg.2021.785892

**Published:** 2022-01-03

**Authors:** Eva Grosfeld, Daan Scheepers, Armin Cuyvers

**Affiliations:** ^1^Europa Institute, Leiden Law School, Leiden University, Leiden, Netherlands; ^2^Department of Psychology, Faculty of Social and Behavioural Sciences, Leiden University, Leiden, Netherlands; ^3^Department of Psychology, Faculty of Social and Behavioural Sciences, Utrecht University, Utrecht, Netherlands

**Keywords:** perceived legitimacy, court of justice, European Union, value alignment, moral foundations

## Abstract

The present study aims to extend research on the role of values for the perceived legitimacy of legal authorities by focusing on (1) supranational legal authorities and (2) a broad range of values. We examine how (alignment between) people’s personal values and their perception of the values of the European Union (EU) are related to perceived legitimacy of the Court of Justice of the EU (CJEU) and the EU more broadly. Inspired by moral foundations theory, we distinguish between individualizing (i.e., “democracy”, “liberty”, and “fairness”) and binding values (i.e., “rule of law”, “respect for national authority”, and “respect for tradition”). An online survey was conducted in six EU member states (*N* = 1,136). A factor analysis confirmed a two-factor model (individualizing vs. binding values) for both personal values and perceived EU values. Four regression models were run for each of the value factors, including personal values, perceived EU values, and their interaction, on each of the outcomes (i.e., perceived CJEU and EU legitimacy). Perceived endorsement by the EU of both individualizing and binding values predicted higher legitimacy perceptions of the CJEU and EU. Furthermore, personal binding values had a negative effect on perceived EU legitimacy when participants perceived the EU to weakly support binding values, but a positive effect when the EU was perceived to strongly support binding values. The results suggest that value alignment plays an important role in perceived legitimacy of the CJEU and EU, and that better representing binding values might be a strategy to improve perceived EU legitimacy.

## Introduction

Although disputed, the perceived legitimacy of the Court of Justice of the European Union (CJEU) is according to some studies declining (Pollack, 2018). Perceived legitimacy can be defined as the belief that an institution exercises its authority appropriately (Tyler, 2006). Analyses of Eurobarometer data suggest that since 2010, following the trend of trust in the European Union (EU) more generally, public trust in the CJEU has declined while distrust has increased (Pollack, 2018). The same may be true for perceptions of CJEU legitimacy among national authorities, who show resistance in terms of non-compliance with CJEU rulings and efforts to limit the effectiveness of CJEU decisions (Hofmann, 2018). Regardless of whether the CJEU’s and EU’s legitimacy levels are actually declining or not, it is generally agreed upon that legal authorities require a widespread basis of perceived legitimacy to maintain social order, settle disputes, and solve societal issues (Tyler and Lind, 1992; Tyler, 2006; Trinkner and Tyler, 2016). Moreover, low legitimacy may have far-reaching consequences for the EU as a whole. For example, taking back control over British law was one of the red lines of the Leave-campaign in the Brexit referendum. It is therefore important to understand when and why people perceive the CJEU and EU as legitimate.

In this brief research report, we examine how people’s personal values, their perceived values of the EU, and alignment between these values relate to public perceived legitimacy of the CJEU and EU. In doing so, we will look beyond “individualizing” values and also consider “binding” values. In what follows, we first discuss theories on how moral judgments may influence the perceived legitimacy of legal authorities, and then elaborate on how individual differences in moral intuitions may explain why some people perceive the CJEU and EU to be legitimate and others do not.

Through interactions with the legal system throughout their lives, individuals develop a relationship with legal authorities. When this relationship is based on the subjective belief that power is exercised appropriately, rather than on fear for punishments, people are more likely to accept the law, even when it goes against their own self-interest (Trinkner and Tyler, 2016). Such legitimacy attributions develop in an ongoing dialogue between power-holders, which claim that their authority and exercise of power are rightful, and members of the “audience”, which process and respond to these claims (Bottoms and Tankebe, 2012).

Legal authorities draw a large part of their legitimacy from “value alignment”, that is, the extent to which the values they endorse align with people’s personal values (Jackson et al., 2012). There are two routes through which value alignment is thought to promote legitimacy. First, shared values communicate to people that they are valued members of the group, which provides them with status and a positive social identity (Tyler and Lind, 1992; Tyler, 1997). As a consequence, personal concerns become less relevant, and people are more likely to internalize the conviction that it is right to obey the rules which are imposed upon them (Tyler and Jackson, 2013). Second, the belief that an authority is acting morally appropriate and in line with one’s own sense of right and wrong normatively validates its power and forms a source of trust (Suchman, 1995; Jackson et al., 2015).

A central way in which *national* legal authorities create value alignment with their audience is through procedural justice. Perceived fair procedures contribute to positive group identification and express the moral appropriateness of authority (Tyler and Blader, 2003; Jackson et al., 2012, 2015). Over the past decades, research has convincingly shown that perceived procedural justice is positively related to the perceived legitimacy of the police, judges, and other court officials (e.g., Sunshine and Tyler, 2003; van den Bos et al., 2014; Tyler et al., 2015; Grootelaar and van den Bos, 2018; see for a meta-analysis Walters and Bolger, 2019). In sum, legal authorities can generate legitimacy by demonstrating value alignment through procedural justice.

However, this is not to say that procedural justice is the only foundation of value alignment. The expression of other values, such as effectiveness or distributive justice, may also justify the exercise of legal power (Bottoms and Tankebe, 2012; Jackson et al., 2015). Where *supranational* authorities are concerned, values such as democracy and transparency, have, for example, been found relevant (Dellmuth et al., 2019). For the legitimacy of the CJEU’s supranational authority, procedural justice may also play a less important role. Although EU law is an integral part of national legal systems, and the CJEU plays a central role in upholding EU law and safeguarding its uniform interpretation and application, lay people seldom interact with the CJEU, and many are even not very aware of its existence (Caldeira and Gibson, 1995). To begin with, the standing of non-privileged parties, such as private individuals, for direct actions to the CJEU is very limited. In addition, the chances of an individual ending up in front of the CJEU via a preliminary reference procedure—in which national courts ask the CJEU for a judgment on the interpretation or validity of EU law within the context of a national dispute—are extremely limited as well (see judicial activity in the annual report of the CJEU, 2020). For these reasons, legitimacy of the CJEU as perceived by the public may not solely rely on procedural justice.

Moreover, when people have no information about the trustworthiness or objective legitimacy of a supranational organization, they are inclined to resort to their feelings about more well-known and visible related authorities, which has been termed the “vertical legitimacy spillover effect” (Haack et al., 2014). Prior research has, for example, shown that the perceived legitimacy of the CJEU is strongly related to the perceived legitimacy of national legal systems (Voeten, 2013) and of the EU in general (Caldeira and Gibson, 1995; Voeten, 2013; Pollack, 2018). People may thus partly base their legitimacy judgments about the CJEU on value alignment with the EU.

In sum, what matters is that people experience a sense of shared values. This in turn depends on which values people *personally* endorse. According to moral foundations theory (MFT; Haidt, 2007), the range of human moral values can be classified into two main categories. On the one hand, there are “individualizing” moral foundations, which are focused on protecting the *individual*. These values are “care” and “fairness.” On the other hand, there are “binding” moral foundations, such as “ingroup loyalty”, “respect for authority”, and “purity”, which are focused on protecting the *group*. While some people are predominantly drawn to individualizing foundations, others are equally or even more drawn to binding foundations (Haidt, 2007). These dispositions in turn have shown to underlie political values and opinions, as political liberals typically only rely on individualizing moral foundations, while political conservatives endorse all five foundations equally (Haidt and Graham, 2007; Graham et al., 2009). For example, the Brexit campaign showed to appeal to all of the public’s moral foundations, which may have influenced votes to leave the EU (Smith, 2019).

In western societies, there is a narrow focus on individualizing moral foundations (Haidt, 2007). Values that resonate strongly with these foundations, such as freedom, equality, and respect for human rights, form the very foundation of the EU and EU law (Article 2 of the Treaty on the European Union). Yet, in these same societies, a large number of people also endorse binding moral foundations (Haidt, 2007). Considering that value alignment constitutes an important element of perceived legitimacy (Jackson et al., 2012) and that individuals have different moral intuitions, it is necessary to look beyond individualizing values when trying to understand perceived legitimacy. For example, people who appreciate tradition and loyalty to their nation may not perceive their values to be particularly represented in EU law, which claims supremacy over even the national constitution.

The present study therefore examines how personal values and perceived values of the EU, as well as alignment between personal and perceived EU values, are related to the perceived legitimacy of the CJEU and EU. These associations are tested for a range of values, which differ in their individualizing (i.e., “democracy”, “liberty”, and “fairness”) versus binding orientation (i.e., “rule of law”, “respect for national authority”, and “respect for tradition”).

## Materials and Methods

### Procedure

We collected data among 1,180 individuals from Finland, France, Germany, Italy, Netherlands, and Poland via the online participant platform Prolific. This data collection comprised multiple measures, of which different parts may appear in future publications. After providing informed consent, participants filled in an online questionnaire, which was designed with Qualtrics software and took approximately 10 min. After that, participants were debriefed and reimbursed with £1. The data and material can be accessed at https://osf.io/6hcw4/?view_only=dfd482abc82548d2afcfd08ae5aaef07. Data collection was ethically approved by the Psychology Research Ethics Committee of the Faculty of Social Sciences at Leiden University (2020-10-22-D.T. Scheepers-V1-2710).

### Participants

After removing data of participants who failed to correctly answer both of two attention checks (*n* = 36), who finished the study in less than 5 minutes (*n* = 6), and whose data were missing (*n* = 2), the total sample consisted of 1,136 participants (*n*_Finland_ = 164, *n*_France_ = 197, *n*_Germany_ = 195, *n*_Italy_ = 200, *n*_Netherlands_ = 196, *n*_Poland_ = 184). The mean age was 27.60 (*SD* = 8.98). Of the participants, 456 identified as female (40.1%), 663 as male (58.4%), 15 as other (1.3%), and 2 did not indicate their gender (0.2%). The sample was on average highly educated, with 29.8, 26.1, and 5.5% having earned, respectively, a university’s bachelor, master, and doctoral degree. Only 3.3% received primary education, whereas 19.5% received secondary education and 15.8% vocational or professional education. The sample was leaning toward the left on the political spectrum (*M* = 28.11, *SD* = 24.46, on a 100-point scale ranging from left/progressive to right/conservative). Most participants were either not very aware (32.9%) or somewhat aware (46.6%) of the CJEU, and only few participants had never heard of it (9.9%) or were very aware of it (10.7%).

### Materials

#### Political Ideology

Political ideology was measured to test whether the values we included in the study followed the individualizing-binding pattern predicted by MFT. We asked participants: “In political matters people talk of ‘the left’ and ‘the right’. How would you place your views on this scale with regard to the economic and social dimension?.” Only responses on the social dimension mattered for the current study. Participants could indicate their social political orientation on a scale from 1 (“Left/progressive”) to 100 (“Right/conservative”).

#### Awareness CJEU

Awareness of the CJEU was measured with one item: “The Court of Justice of the European Union sits in Luxembourg and is the highest court of the European Union as a whole. How aware would you say you are of this court?,” with four answer options: *never heard of it before now*, *not very aware*, *somewhat aware*, and *very aware*.

#### Values and Value Alignment

The perceived values of the EU were measured with the question: “To what extent do you consider each of these values to be endorsed by the European Union?,” for each of the values: “Democracy”, “Liberty”, “Fairness”, “Rule of law (e.g., respect for independence of the judiciary, the integrity and impartiality of the electoral system)”, “Respect for national authority”, and “Respect for tradition.” Answers were provided on 5-point Likert scales ranging from *not at all endorsed* to *extremely endorsed*. After measuring EU values, we also measured personal values by asking participants to indicate for the same values: “How important are each of these values to yourself?,” using 5-point Likert scales ranging from *not at all important* to *extremely important*. Value alignment was operationalized as overlap between personal values and perceived EU values.

#### Perceived Legitimacy

The perceived legitimacy of the CJEU and the EU was operationalized as institutional trust and felt duty to obey. We adapted items from previous work that measured the perceived legitimacy of the police (Sunshine and Tyler, 2003; Jackson et al., 2012), resulting in a total scale of nine items (α_CJEU_ = 0.95, α_EU_ = 0.93; e.g., “I have confidence in the CJEU [EU],” “People should obey decisions from the CJEU [laws made by the EU] even if they will not be caught for breaking them”), which were answered on 7-point Likert scales ranging from *strongly disagree* to *strongly agree*.

### Data Analysis

The data were analyzed in RStudio (Version 1.3.959). After descriptive analyses, we performed a maximum-likelihood Confirmatory Factor Analysis (CFA) for the set of personal values and the set of perceived EU values, where individualizing values and binding values were specified as two separate factors. Then, two times two ordinary least squares (OLS) regression models with fixed effects were run for each of the value factors (i.e., individualizing values and binding values) on each of the outcome variables (i.e., perceived legitimacy of the CJEU and perceived legitimacy of the EU). The models controlled for country, age, and education. Predictor variables were personal endorsement of [individualizing-binding] values, perceived endorsement by the EU of [individualizing-binding] values, and the interaction term between [individualizing-binding] values, which was used as an indicator of value alignment. All continuous variables were mean centered.

## Results

**Figure 1:**
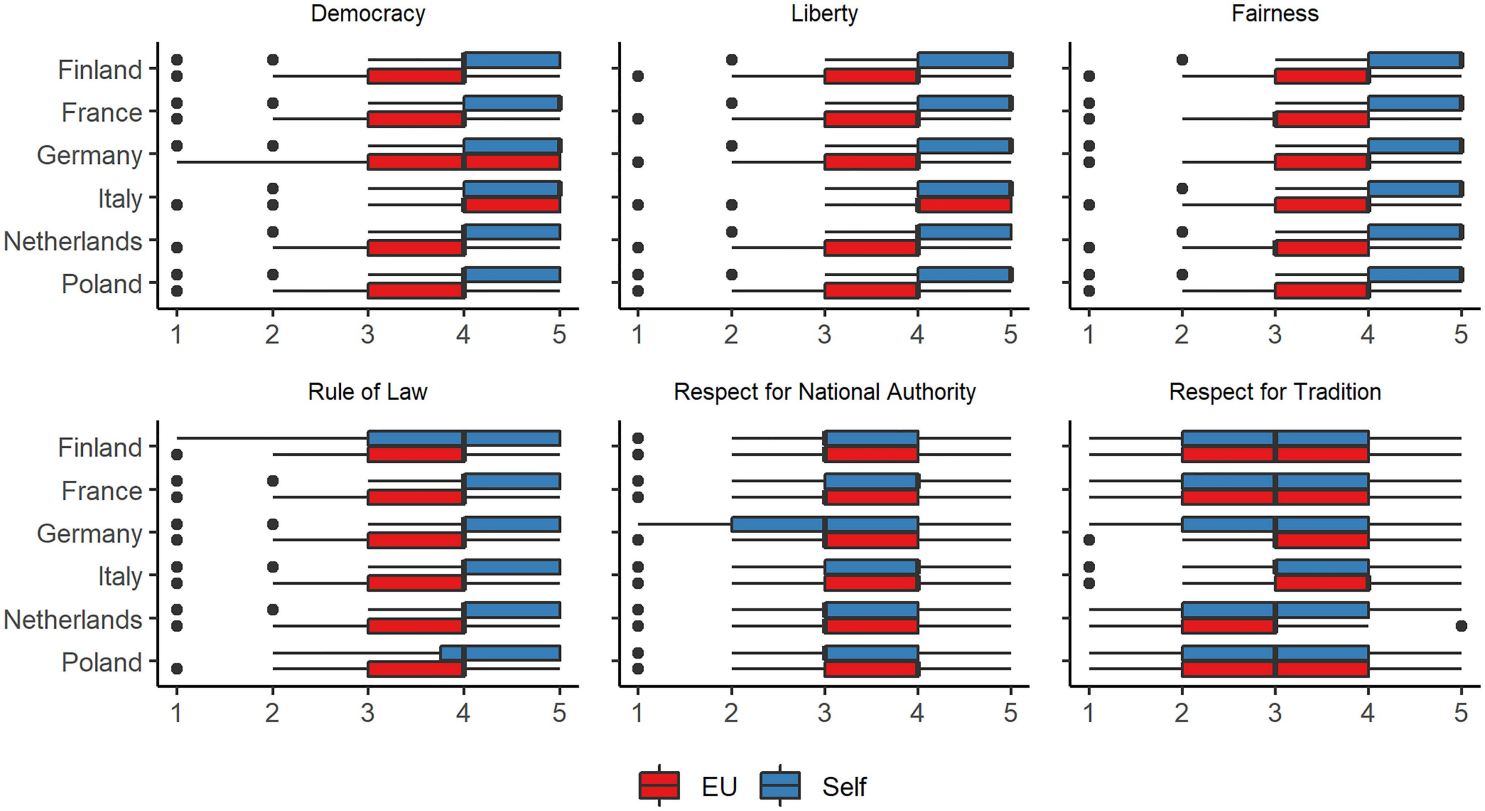
The distribution of personal values and perceived values of the EU in all country subsamples.

Figure 1 presents the distribution of personal values and perceived EU values for each value separately and in each of the country subsamples. With regard to both the personal and perceived EU values democracy, liberty, fairness, and rule of law, the data were left-skewed, indicating that most participants highly supported these values and also perceived the EU to support these values. Respect for national authority and respect for tradition were more evenly distributed among all samples, both regarding personal endorsement and perceived endorsement by the EU. Means, standard deviations, and skewness scores are reported in Supplementary Table S1.

**Figure 2:**
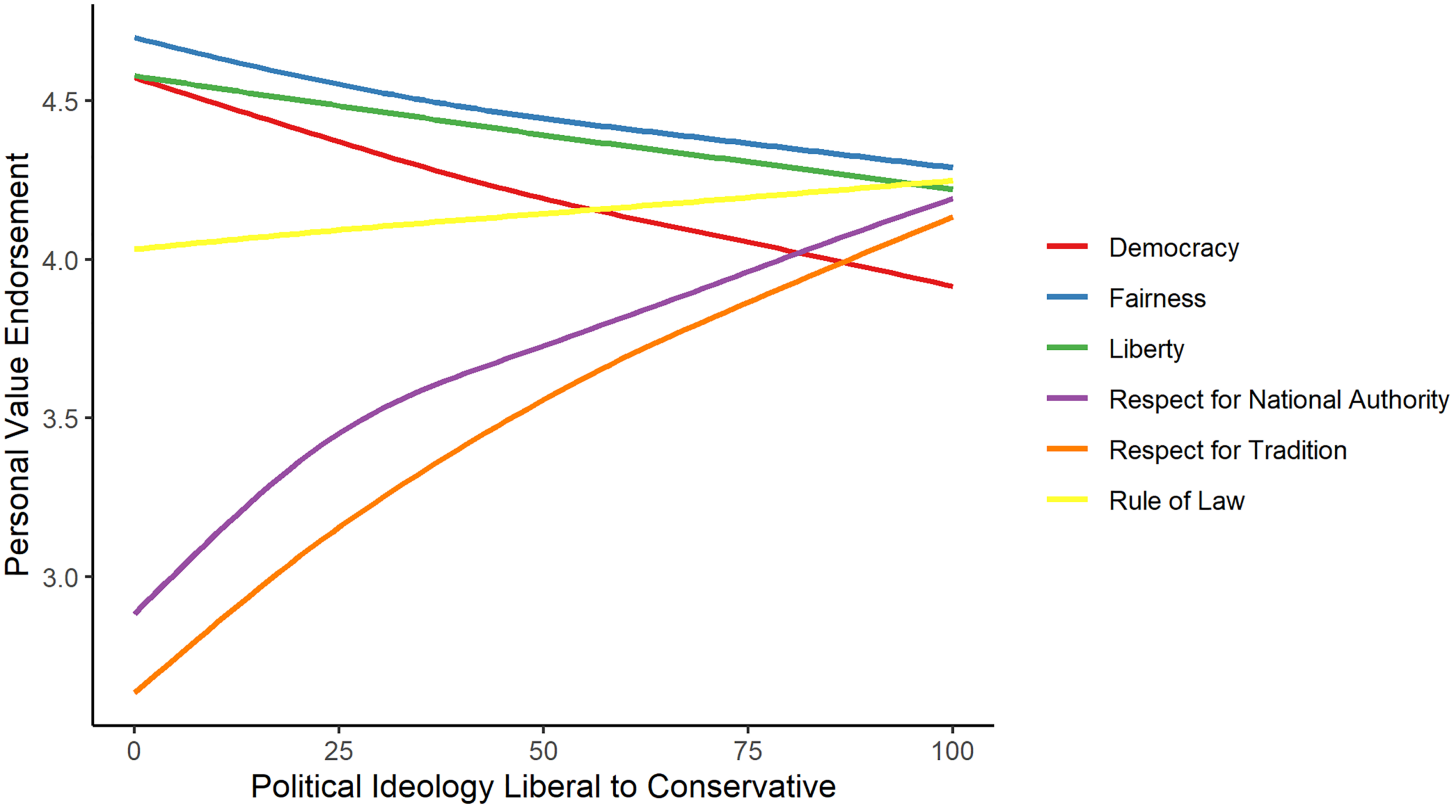
The relation between personal values and political ideology.

Figure 2 shows the relation between each of the personal values and political ideology. Pearson correlations showed that more right-oriented political ideology was related to lower scores on individualizing values (*r* = −0.23, *p* < 0.001) and higher scores on binding values (*r* = 0.33, *p* < 0.001). An OLS regression model in which personal values were regressed on political ideology, controlling for demographic variables (age, gender, and education), confirmed that political ideology was predicted by personal values. More specifically, a more right-oriented political ideology was positively predicted by respect for national authority (*b* = 4.71, *SE* = 0.74, *p* < 0.001) and respect for tradition (*b* = 4.46, *SE* = 0.62, *p* < 0.001), and negatively predicted by democracy (*b* = −4.98, *SE* = 0.87, *p* < 0.001), liberty (*b* = −2.74, *SE* = 0.97, *p* = 0.005), and fairness (*b* = −2.92, *SE* = 1.02, *p* = 0.004). Personal endorsement of the rule of law did not predict political ideology (*b* = 0.31, *SE* = 0.83, *p* = 0.705).

Both the CFA on personal values and the CFA on perceived EU values revealed a good fit for the two specified factors, where democracy, liberty, and fairness loaded on “individualizing values” and rule of law, respect for national authority, and respect for tradition loaded on “binding values” (see Supplementary Tables S2 and S3).^1^ The analyses were therefore continued with the factors.

Table 1 shows descriptive statistics and Pearson correlations between the variables. The average perceived legitimacy of the CJEU and EU were both relatively high, with the former being still somewhat higher than the latter, Δ_means_ = 0.39, 95% CI [0.30, 0.49], *t*(2270) = 8.18, *p* < 0.001. Legitimacy perceptions of both institutions were highly correlated. Moreover, for both individualizing and binding values, the CJEU and EU were perceived as more legitimate when participants personally endorsed these values and when they perceived the EU to endorse them.

**Table 1:** Descriptive statistics and Pearson correlations between study variables.

	1	2	3	4	5	6	7	8	9	10
1. Age										
2. Education	0.20^***^									
3. Political ideology	0.11^***^	−0.09^**^								
4. Awareness CJEU	0.12^***^	0.15^***^	−0.03							
5. Pers. ind. Values	0.10^***^	0.16^***^	−0.23^***^	0.13^***^						
6. Pers. bind. Values	0.09^**^	0.02	0.33^***^	0.12^***^	0.19^***^					
7. Perc. ind. Values EU	−0.07^*^	0.09^**^	−0.22^***^	0.12^***^	0.30^***^	0.11^***^				
8. Perc. bind. Values EU	−0.07^*^	0.03	−0.21^***^	0.09^**^	0.21^***^	0.15^***^	0.67^***^			
9. Perc. legitimacy CJEU	0.12^***^	0.24^***^	−0.18^***^	0.23^***^	0.22^***^	0.12^***^	0.51^***^	0.46^***^		
10. Perc. legitimacy EU	<0.01	0.17^***^	−0.24^***^	0.13^***^	0.18^***^	0.09^**^	0.61^***^	0.56^***^	0.80^***^	
*M*	27.60	–	28.11	2.58	4.46	3.55	3.65	3.36	5.02	4.62
*SD*	8.98	–	24.46	0.81	0.55	0.82	0.87	0.85	1.15	1.15

*Pers. = personal; perc. = perceived; ind. = individualizing; and bind. = binding*.

^*^p < 0.05, ^**^p < 0.01, and ^***^p < 0.001.

The regression model with individualizing values on perceived legitimacy of the CJEU showed that perceived individualizing values of the EU were a significant positive predictor of perceived CJEU legitimacy, *b* = 0.49, *SE* = 0.03, 95% CI [0.43, 0.54], *p* < 0.001, indicating that the more participants perceived the EU to endorse individualizing values, the higher the perceived legitimacy of the CJEU. The regression model with binding values showed a similar effect of perceived binding values of the EU on perceived legitimacy of the CJEU, *b* = 0.44, *SE* = 0.03, 95% CI [0.38, 0.49], *p* < 0.001. Perceived legitimacy of the CJEU was not predicted by personal individualizing-binding values nor by the interaction between personal values and perceived EU values (see Supplementary Tables S4 and S5 for the results of these models).

The model with individualizing values on perceived legitimacy of the EU showed that perceived individualizing values of the EU were a significant positive predictor, *b* = 0.60, *SE* = 0.03, 95% CI [0.55, 0.65], *p* < 0.001. This suggests that perceived legitimacy of the EU is higher when people perceive the EU to endorse individualizing values. The model with binding values also showed a significantly positive effect of perceived binding values of the EU on perceived EU legitimacy, *b* = 0.52, *SE* = 0.03, 95% CI [0.47, 0.58], *p* < 0.001. This model in addition revealed a positive, significant interaction between personal binding values and perceived binding values of the EU, *b* = 0.07, *SE* = 0.02, 95% CI [0.02, 0.11], *p* = 0.002 (see Supplementary Tables S6 and S7 for the results of these models). Simple slope analyses were conducted to better understand this interaction. These showed that personal binding values had a significantly negative effect on perceived EU legitimacy when participants perceived that the EU weakly represents binding values [1 *SD* below mean; *β* = −0.06, *SE* = 0.03, 95% CI (−0.12, 0), *p* = 0.070], but that this effect was positive when participants perceived that the EU strongly represents binding values [*β* = 0.08, *SE* = 0.04, 95% CI (0.01, 0.15), *p* = 0.030].

**Figure 3:**
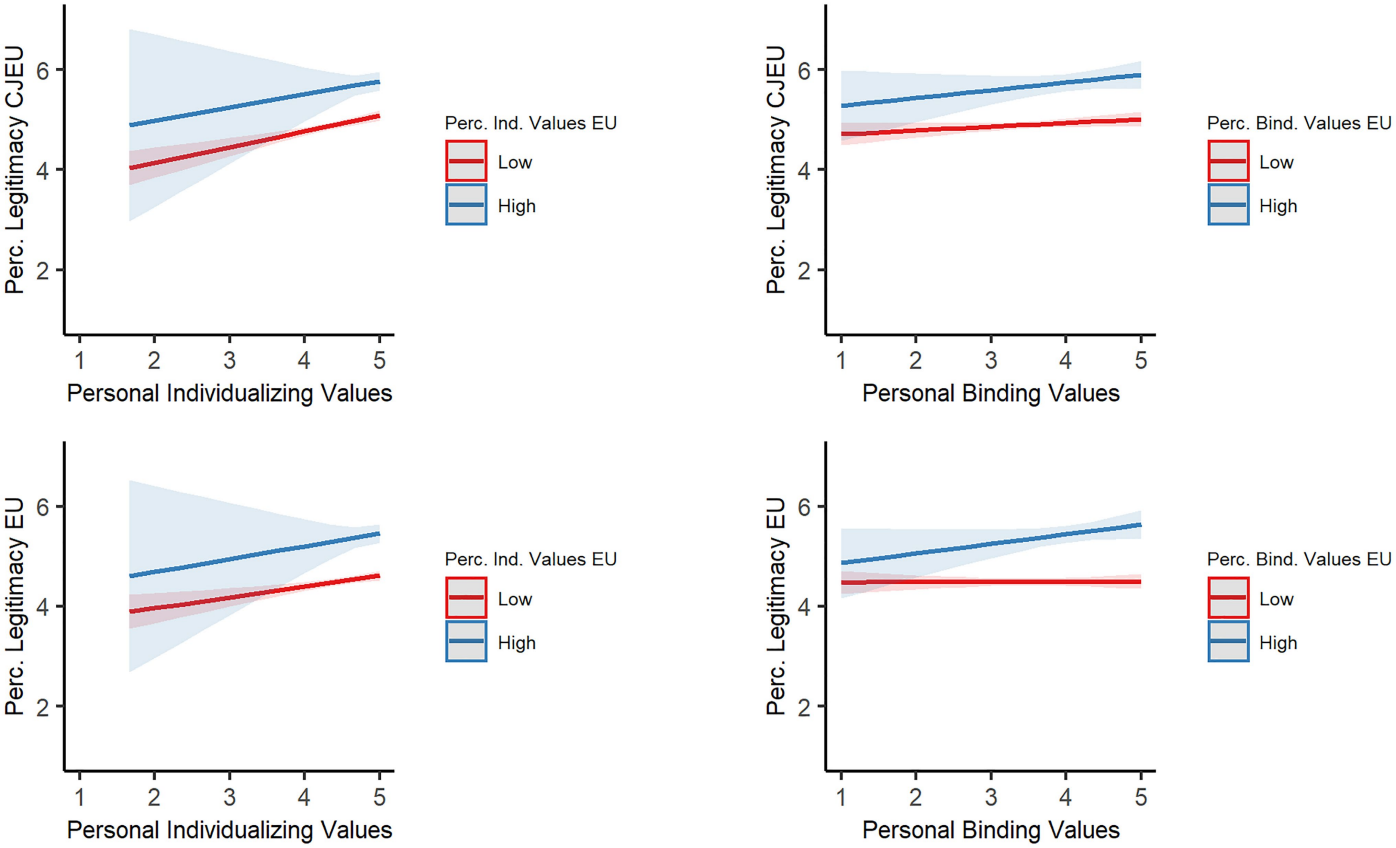
Slopes of the interaction between personal values and perceived values of the EU on perceived legitimacy of the CJEU and EU.

In sum, as illustrated in Figure 3, the CJEU and EU were perceived as more legitimate when the EU’s endorsement of individualizing and binding values was high (1 *SD* above mean) versus low (1 *SD* below mean). The bottom-right panel of Figure 3 shows that the positive effect of EU binding values on perceived EU legitimacy was qualified by an interaction with personal binding values. This interaction entails that personal binding values were unrelated to perceived EU legitimacy when the EU was perceived to weakly endorse binding values (red line); however, personal binding values predicted perceived EU legitimacy when the EU was perceived to strongly support them (blue line).

## Discussion

How do people come to perceive the CJEU and EU as (il)legitimate? Understanding these processes is important for the effectiveness and viability of these legal institutions, especially now that the rule of law crisis in *inter alia* Poland and Hungary openly challenges the CJEU’s power to uphold EU law in the face of national opposition. Prior research has proposed that alignment between the values of a legal authority and its audience is a key source of perceived legitimacy, but this work has predominantly focused on national legal authorities and procedural justice (Jackson et al., 2012, 2015; Tyler and Jackson, 2013). In the present study, we extended these findings to the supranational level and explored which other values are relevant to the perceived legitimacy of the CJEU and EU. As the EU is a diverse society, comprising multiple countries and cultures, we investigated how perceived legitimacy of the CJEU and EU is related to both individualizing (i.e., democracy, liberty, and fairness) and binding values (i.e., rule of law, respect for national authority, and respect for tradition).

The findings showed that legitimacy perceptions of the CJEU and EU are, after controlling for demographic variables, higher when people perceive the EU to support both individualizing and binding values. Except for one model, we found no interactions between personal values and perceived EU values. However, although we conceptualized value alignment as the interaction between personal values and perceived EU values, we would not argue that these results should be interpreted as meaning that value alignment is not a source of perceived legitimacy. After all, there was little variation on personal individualizing values scores, showing that all participants strongly supported democracy, liberty, and fairness (i.e., a ceiling effect). Considering the positive effect of perceived endorsement of these values by the EU on perceived legitimacy of the CJEU and EU, it could be argued that value alignment with regard to these values is a source of legitimacy, but that people with individualizing moral foundations find their values already represented by the EU.

For the model where we did find an interaction, i.e., the model where binding values were regressed on perceived legitimacy of the EU, scores on personal values were more evenly distributed across participants. Here, the results revealed that legitimacy decreased as personal support for binding values increased and that this effect was neutralized and actually reversed into a positive effect when participants believed that the EU also supports binding values. These findings are indicative of a value alignment effect for binding values, implying that when the EU fails to serve people with binding moral foundations, the EU may be perceived as less legitimate by these people. Practically, these findings may imply that when the EU better represents binding values, in addition to individualizing values, there will be a positive effect on perceived legitimacy of the EU.

Furthermore, the findings indicated that the CJEU is still not widely known among the public. Awareness increased with higher education but was unrelated to political ideology. As suggested by the high correlation between perceived legitimacy of the CJEU and EU, people’s conferral of legitimacy to the CJEU is partly derived from their feelings toward the EU. This is consistent with the vertical legitimacy spill-over effect, which holds that people use affect heuristics to judge the legitimacy of a transnational authority (Haack et al., 2014). Although we cannot say with certainty that perceived values of the EU causally spill over to the perceived legitimacy of the CJEU, the findings provide correlational evidence to suggest that this effect also applies to international courts.

The findings should be interpreted while noting the study’s limitations. First, our sample included people from only six member states, who may have represented a certain social class as they were required to be fluent in English, limiting generalizability of the findings. In addition, due to the study’s cross-sectional nature, the results cannot give insight into causality. Furthermore, we based our expectations about the differences between individualizing and binding values on MFT but did not measure the “traditional” moral foundations. Although the values that were included in our study were closely related to the values of MFT and followed a similar pattern with regard to political ideology, it would nevertheless be interesting to see whether the results remain when using the traditional MFT items. The value of freedom, moreover, may represent a sixth moral foundation, which has been identified as not belonging to the individualizing or binding moral foundations: liberty, which is characterized by strong endorsement of individual liberty and resentment of any sign of domination or repression (Iyer et al., 2012). Follow-up studies could look into when and how freedom/liberty is relevant for perceived legitimacy of the EU. For example, by studying how political parties’ framing of freedom affects perceived legitimacy, as it could be framed as “the freedom of minority groups to make individual choices without oppression from majority elites” but also as “the freedom to decide for ‘ourselves’ without interference from ‘Brussels’.”

Finally, no scholarly consensus exists about the meaning of perceived legitimacy and the best way to measure it. Here, we operationalized perceived legitimacy as institutional trust and felt duty to obey (Sunshine and Tyler, 2003). However, legitimacy, trust, and duty to obey may overlap and differ, and can differently affect law-related behavior (Jackson and Gau, 2016). It is therefore important that future research finds novel ways to measure perceived legitimacy, for example, with behavioral measures, which would also improve our conceptual understanding of perceived legitimacy (*cf.*, Dellmuth and Schlipphak, 2020).

As for other future directions, future research should test the causal directions between value alignment, identification with the EU, and perceived legitimacy of the EU in more controlled lab experiments. Another direction is to examine whether it matters how *effective* the EU is in the eyes of the public at safeguarding their values, since effectiveness in achieving policy objectives has been defined as an institutional source of legitimacy (Dellmuth et al., 2019). Finally, it would be interesting to take into account and better understand the different discourses that may lead to perceived (il)legitimacy of the CJEU and EU, since Euroscepticism can be rooted in different concerns and narratives (Baldassari et al., 2020).

Notwithstanding these limitations, our study sheds light on social psychological processes that lead to perceived legitimacy of the CJEU and EU, highlighting the role of value alignment. Although individualizing values are important to protect, our findings suggest that improving these values may not result in a net increase of perceived legitimacy. Instead, they suggest that some citizens find it equally important that the EU respects binding values, such as respect for tradition and national authority, and that these values are currently not perceived by the public as sufficiently safeguarded by the EU. Better serving people with binding values could therefore be a strategy to improve perceived legitimacy of the CJEU and EU. Of course, this perspective brings new legal and political difficulties, for it is harder for authorities to represent everyone’s values in multicultural societies (*cf.*, Tyler and Jackson, 2013). However, this only underlines the demand for a better understanding of the potential and pitfalls of value alignment for perceived legitimacy of the CJEU and EU.

## Data Availability Statement

The datasets presented in this study can be found in online repositories. The names of the repository/repositories and accession number(s) can be found at: https://osf.io/6hcw4/?view_only=dfd482abc82548d2afcfd08ae5aaef07.

## Ethics Statement

The studies involving human participants were reviewed and approved by the Psychology Research Ethics Committee, Leiden University (2020-10-22-D.T. Scheepers-V1-2710). The patients/participants provided their written informed consent to participate in this study.

## Author Contributions

All authors discussed the objectives of the data generated for this study, which is part of a larger research project, and the structure of the paper. EG analyzed the data and wrote the manuscript. DS and AC reviewed and edited the manuscript.

## Funding

The current research was funded by Leiden Law School, Leiden University, through Dutch national sector plan for law, theme: “Institutions for Conflict Resolution.”

## Conflict of Interest

The authors declare that the research was conducted in the absence of any commercial or financial relationships that could be construed as a potential conflict of interest.

## Publisher’s Note

All claims expressed in this article are solely those of the authors and do not necessarily represent those of their affiliated organizations, or those of the publisher, the editors and the reviewers. Any product that may be evaluated in this article, or claim that may be made by its manufacturer, is not guaranteed or endorsed by the publisher.

## Footnotes

^1^As also shown in Supplementary Tables S3 and S4, the rule of law loaded on both factors, and regarding perceived EU values, the loading on individualizing values was even higher than on binding values. This is not surprising, since the rule of law could be reasonably considered a value which not only relates to binding values, but also to individualizing values—breaches of the rule of law have even become illustrative for conservative governments in Poland and Hungary, which may enhance the significance of this value for people with individualizing values who are often more liberal.

## Supplementary Material

The Supplementary Material for this article can be found online at: https://www.frontiersin.org/articles/10.3389/fpsyg. 2021.785892/full#supplementary-material
