# Supplementary material for: Value Alignment and Public Perceived Legitimacy of the European Union and the Court of Justice
Source: Front Psychol. 2022 Jan 3;12:785892. doi: 10.3389/fpsyg.2021.785892 (PMC8761663; doi:10.3389/fpsyg.2021.785892)
Supplement: Supplementary file 1 [file Table_1.docx]

Supplementary Material

# Supplementary Tables

**Supplementary Table 1.** Descriptive statistics of personal values and perceived EU values

|  | *M* | *SD* | Skewness |
| --- | --- | --- | --- |
| Personal values |  |  |  |
| Democracy | 4.36 | 0.71 | -1.41 |
| Liberty | 4.47 | 0.69 | -1.33 |
| Fairness | 4.55 | 0.69 | -1.70 |
| Rule of law | 4.10 | 0.87 | -0.82 |
| Respect for national authority | 3.40 | 1.09 | -0.29 |
| Respect for tradition | 3.16 | 1.21 | -0.07 |
| Perceived EU values |  |  |  |
| Democracy | 3.80 | 0.99 | -0.89 |
| Liberty | 3.70 | 0.97 | -0.67 |
| Fairness | 3.44 | 1.04 | -0.44 |
| Rule of law | 3.67 | 0.99 | -0.62 |
| Respect for national authority | 3.27 | 1.02 | -0.43 |
| Respect for tradition | 3.13 | 1.08 | -0.13 |

**Supplementary Table 2.** Results from a factor analysis of personal values

| Value | Factor loading | |  |
| --- | --- | --- | --- |
|  | 1 | 2 | Eigenvalue |
| Factor 1: Individualizing values |  |  | 1.19 |
| Democracy | **.59** |  |  |
| Liberty | **.56** |  |  |
| Fairness | **.63** |  |  |
| Factor 2: Binding values |  |  | 1.45 |
| Rule of law | .35 | **.40** |  |
| Respect for national authority |  | **.98** |  |
| Respect for tradition |  | **.57** |  |

*Note*. Factor loadings above .35 are in bold. The two-factor model had a good fit, χ^2^(8) = 97.39, *p* < .001; CFI = .921; TLI = .852; RMSEA = .099; SRMR = .070.

**Supplementary Table 3.** Results from a factor analysis of perceived EU values

| Value | Factor loading | |  |
| --- | --- | --- | --- |
|  | 1 | 2 | Eigenvalue |
| Factor 1: Individualizing values |  |  | 2.25 |
| Democracy | **.77** | .25 |  |
| Liberty | **.77** | .29 |  |
| Fairness | **.70** | .27 |  |
| Factor 2: Binding values |  |  | 1.61 |
| Rule of law | **.60** | **.40** |  |
| Respect for national authority | .28 | **.96** |  |
| Respect for tradition | .35 | **.56** |  |

*Note*. Factor loadings above .35 are in bold. The two-factor model had a good fit, χ^2^(8) = 186.22, *p* < .001; CFI = .943; TLI = .893; RMSEA = .140; SRMR = .050.

**Supplementary Table 4.** Results from regression analyses on perceived legitimacy of the CJEU with individualizing values

| Effect | Estimate | | *SE* | | 95% CI | | | | *p* | | VIF |
| --- | --- | --- | --- | --- | --- | --- | --- | --- | --- | --- | --- |
|  |  | |  | | *LL* | | *UL* | |  | |  |
| Intercept | -0.01 | 0.15 | | -0.30 | | 0.28 | | .954 | |  | |
| Country^a^ |  |  | |  | |  | |  | | 1.53 | |
| France | -0.42 | 0.09 | | -0.60 | | -0.24 | | < .001 | |  | |
| Germany | -0.09 | 0.09 | | -0.26 | | 0.09 | | .323 | |  | |
| Italy | -0.18 | 0.09 | | -0.36 | | 0.00 | | .052 | |  | |
| Netherlands | -0.02 | 0.09 | | -0.20 | | 0.15 | | .784 | |  | |
| Poland | -0.22 | 0.09 | | -0.40 | | -0.04 | | .020 | |  | |
| Age | 0.10 | 0.03 | | 0.05 | | 0.15 | | < .001 | | 1.15 | |
| Education^b^ |  |  | |  | |  | |  | | 1.46 | |
| Secondary education | -0.06 | 0.15 | | -0.35 | | 0.22 | | .662 | |  | |
| Vocational education | -0.20 | 0.17 | | -0.54 | | 0.14 | | .256 | |  | |
| Professional education | 0.15 | 0.16 | | -0.16 | | 0.47 | | .346 | |  | |
| Bachelor’s degree | 0.20 | 0.15 | | -0.09 | | 0.48 | | .173 | |  | |
| Master’s degree | 0.37 | 0.15 | | 0.08 | | 0.66 | | .011 | |  | |
| Doctoral degree | 0.42 | 0.18 | | 0.08 | | 0.77 | | .016 | |  | |
| Pers. ind. values | 0.04 | 0.03 | | -0.02 | | 0.09 | | .187 | | 1.32 | |
| Perc. ind. values EU | 0.49 | 0.03 | | 0.43 | | 0.54 | | < .001 | | 1.22 | |
| Pers. ind. Values X perc. ind. values EU | -0.02 | 0.03 | | -0.07 | | 0.04 | | .533 | | 1.16 | |

*Note*. CI = confidence interval; *LL* = lower limit; *UL* = upper limit; VIF = variance inflation factor; pers. = personal; perc. = perceived; ind. = individualizing. *R*^2^ = .329.

^a^ Reference group = Finland. ^b^ Reference group = primary education.

**Supplementary Table 5.** Results from regression analyses on perceived legitimacy of the CJEU with binding values

| Effect | Estimate | | *SE* | | 95% CI | | | | *p* | | VIF |
| --- | --- | --- | --- | --- | --- | --- | --- | --- | --- | --- | --- |
|  |  | |  | | *LL* | | *UL* | |  | |  |
| Intercept | -0.07 | 0.15 | | -0.36 | | 0.23 | | .652 | |  | |
| Country^a^ |  |  | |  | |  | |  | | 1.53 | |
| France | -0.43 | 0.09 | | -0.61 | | -0.25 | | < .001 | |  | |
| Germany | -0.14 | 0.09 | | -0.26 | | 0.09 | | .125 | |  | |
| Italy | -0.20 | 0.09 | | -0.32 | | 0.04 | | .031 | |  | |
| Netherlands | -0.03 | 0.09 | | -0.21 | | 0.15 | | .742 | |  | |
| Poland | -0.30 | 0.10 | | -0.49 | | -0.11 | | .002 | |  | |
| Age | 0.09 | 0.03 | | 0.04 | | 0.14 | | < .001 | | 1.15 | |
| Education^b^ |  |  | |  | |  | |  | | 1.45 | |
| Secondary education | -0.02 | 0.15 | | -0.32 | | 0.28 | | .906 | |  | |
| Vocational education | -0.16 | 0.18 | | -0.51 | | 0.19 | | .381 | |  | |
| Professional education | 0.18 | 0.17 | | -0.15 | | 0.50 | | .288 | |  | |
| Bachelor’s degree | 0.30 | 0.15 | | 0.01 | | 0.59 | | .043 | |  | |
| Master’s degree | 0.49 | 0.15 | | 0.19 | | 0.79 | | .001 | |  | |
| Doctoral degree | 0.53 | 0.18 | | 0.17 | | 0.88 | | .003 | |  | |
| Pers. bind. values | 0.05 | 0.03 | | 0.00 | | 0.10 | | .070 | | 1.09 | |
| Perc. bind. values EU | 0.44 | 0.03 | | 0.38 | | 0.49 | | < .001 | | 1.17 | |
| Pers. bind. values X perc. bind. values EU | 0.03 | 0.02 | | -0.02 | | 0.07 | | .251 | | 1.13 | |

*Note*. CI = confidence interval; *LL* = lower limit; *UL* = upper limit; VIF = variance inflation factor; pers. = personal; perc. = perceived; bind. = binding. *R*^2^ = .291.

^a^ Reference group = Finland. ^b^ Reference group = primary education.

**Supplementary Table 6.** Results from regression analyses on perceived legitimacy of the EU with individualizing values

| Effect | Estimate | | *SE* | | 95% CI | | | | *p* | | VIF |
| --- | --- | --- | --- | --- | --- | --- | --- | --- | --- | --- | --- |
|  |  | |  | | *LL* | | *UL* | |  | |  |
| Intercept | 0.11 | 0.14 | | -0.17 | | 0.38 | | .439 | |  | |
| Country^a^ |  |  | |  | |  | |  | | 1.53 | |
| France | -0.35 | 0.09 | | -0.51 | | -0.18 | | < .001 | |  | |
| Germany | -0.11 | 0.08 | | -0.28 | | 0.05 | | .189 | |  | |
| Italy | -0.11 | 0.09 | | -0.28 | | 0.06 | | .202 | |  | |
| Netherlands | -0.05 | 0.08 | | -0.21 | | 0.12 | | .582 | |  | |
| Poland | -0.27 | 0.09 | | -0.45 | | -0.10 | | .002 | |  | |
| Age | 0.02 | 0.02 | | -0.03 | | 0.06 | | .525 | | 1.15 | |
| Education^b^ |  |  | |  | |  | |  | | 1.46 | |
| Secondary education | -0.08 | 0.14 | | -0.36 | | 0.19 | | .558 | |  | |
| Vocational education | -0.30 | 0.16 | | -0.62 | | 0.03 | | .071 | |  | |
| Professional education | -0.01 | 0.15 | | -0.31 | | 0.29 | | .949 | |  | |
| Bachelor’s degree | 0.08 | 0.14 | | -0.19 | | 0.35 | | .549 | |  | |
| Master’s degree | 0.18 | 0.14 | | -0.09 | | 0.46 | | .186 | |  | |
| Doctoral degree | 0.21 | 0.17 | | -0.12 | | 0.53 | | .211 | |  | |
| Pers. ind. values | -0.02 | 0.13 | | -0.08 | | 0.03 | | .362 | | 1.32 | |
| Perc. ind. values EU | 0.60 | 0.03 | | 0.55 | | 0.65 | | < .001 | | 1.22 | |
| Pers. ind. values X perc. ind. values EU | -0.03 | 0.03 | | -0.07 | | 0.02 | | .322 | | 1.16 | |

*Note*. CI = confidence interval; *LL* = lower limit; *UL* = upper limit; VIF = variance inflation factor; pers. = personal; perc. = perceived; ind. = individualizing. *R*^2^ = .402.

^a^ Reference group = Finland. ^b^ Reference group = primary education.

**Supplementary Table 7.** Results from regression analyses on perceived legitimacy of the EU with binding values

| Effect | Estimate | *SE* | | 95% CI | | | | *p* | | VIF |
| --- | --- | --- | --- | --- | --- | --- | --- | --- | --- | --- |
|  |  |  | | *LL* | | *UL* | |  | |  |
| Intercept | 0.06 | | 0.14 | | -0.22 | | 0.35 | | .660 |  |
| Country^a^ |  | |  | |  | |  | |  | 1.53 |
| France | -0.38 | | 0.09 | | -0.55 | | -0.20 | | < .001 |  |
| Germany | -0.20 | | 0.09 | | -0.37 | | -0.03 | | .021 |  |
| Italy | -0.16 | | 0.09 | | -0.33 | | 0.02 | | .075 |  |
| Netherlands | -0.05 | | 0.09 | | -0.22 | | 0.12 | | .590 |  |
| Poland | -0.35 | | 0.09 | | -0.53 | | -0.17 | | <.001 |  |
| Age | 0.00 | | 0.03 | | -0.05 | | 0.05 | | .946 | 1.15 |
| Education^b^ |  | |  | |  | |  | |  | 1.45 |
| Secondary education | -0.05 | | 0.14 | | -0.33 | | 0.23 | | .720 |  |
| Vocational education | -0.26 | | 0.17 | | -0.60 | | 0.07 | | .122 |  |
| Professional education | 0.01 | | 0.16 | | -0.30 | | 0.32 | | .956 |  |
| Bachelor’s degree | 0.17 | | 0.14 | | -0.10 | | 0.45 | | .221 |  |
| Master’s degree | 0.30 | | 0.14 | | 0.01 | | 0.58 | | .041 |  |
| Doctoral degree | 0.27 | | 0.17 | | -0.06 | | 0.61 | | .110 |  |
| Pers. bind. values | 0.01 | | 0.03 | | -0.04 | | 0.06 | | .640 | 1.09 |
| Perc. bind. values EU | 0.52 | | 0.03 | | 0.47 | | 0.58 | | < .001 | 1.17 |
| Pers. bind. values X perc. bind. values EU | 0.07 | | 0.02 | | 0.02 | | 0.11 | | .002 | 1.13 |

*Note*. CI = confidence interval; *LL* = lower limit; *UL* = upper limit; VIF = variance inflation factor; pers. = personal; perc. = perceived; bind. = binding. R^2^ = .358.

^a^ Reference group = Finland. ^b^ Reference group = primary education.
